# Supplementary material for: Library transgenesis in zebrafish through delayed site-specific mosaic integration for in vivo pooled screening of transgenes
Source: bioRxiv. 2026 Jan 31:2026.01.30.702415. Preprint. [Version 1] doi: 10.64898/2026.01.30.702415 (PMC12873921; doi:10.64898/2026.01.30.702415)
Supplement: 1 [file NIHPP2026.01.30.702415V1-supplement-1.pdf]

bioRxiv preprint doi: https://doi.org/10.64898/2026.01.30.702415; this version posted January 31, 2026. The copyright holder for this preprint (which was not certified by peer review) is the author/funder, who has granted bioRxiv a license to display the preprint in perpetuity. It is made available under aCC-BY 4.0 International license.

Supplementary Information (SI files)

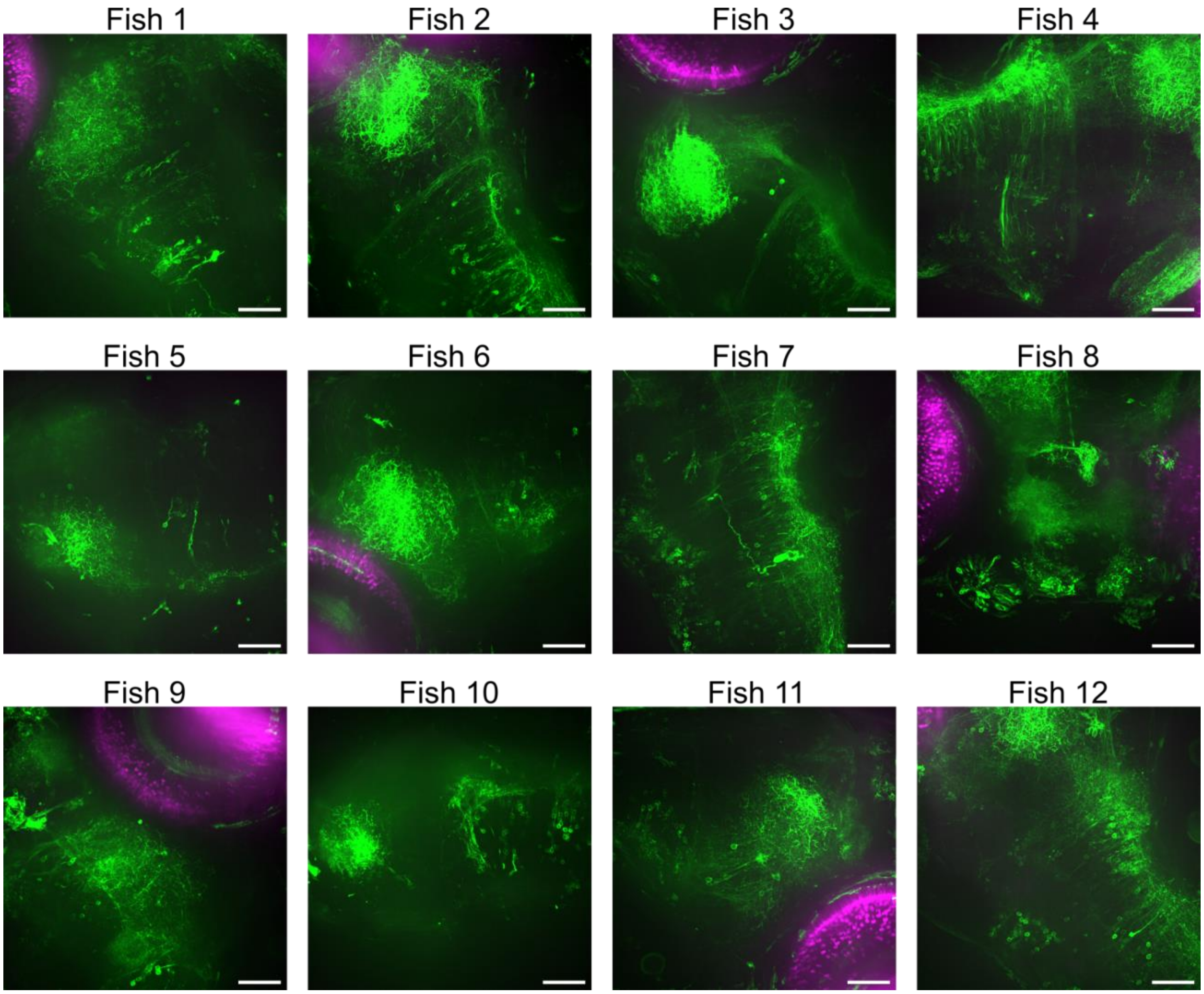

Figure S1 - GFP-CAAX expression in the 12 fish with DNA barcodes characterized by deep sequencing (related to **Fig. 3**). Images presented are max projections from confocal fluorescence imaging of the brain around the optic tectum and/or hindbrain of the animals, with skin autofluorescence removed with manual masks to aid visualization. Magenta fluorescence corresponds to red eye marker expression in the HuC::Gal4;nacre;RH1::DsRed driver fish line. Scale bar = 50 μm.

bioRxiv preprint doi: https://doi.org/10.64898/2026.01.30.702415; this version posted January 31, 2026. The copyright holder for this preprint (which was not certified by peer review) is the author/funder, who has granted bioRxiv a license to display the preprint in perpetuity. It is made available under aCC-BY 4.0 International license.

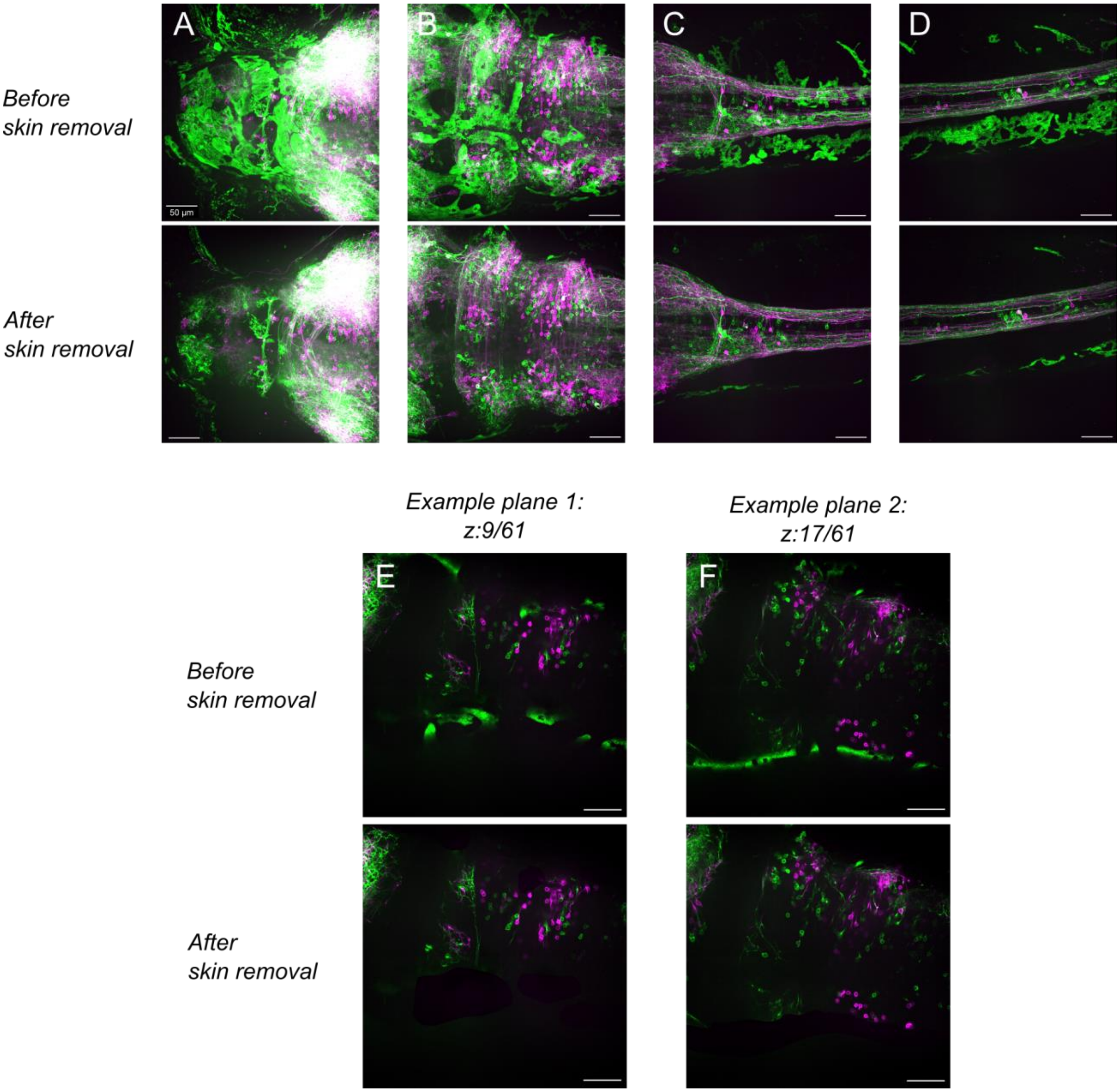

Figure S2 - Demonstration of the skin autofluorescence removal from the max-projections images shown in Fig. 2 and S1. Green autofluorescence from the skin was removed using manually-drawn masks on the individual z-planes, before generating the max-intensity projections. This was done to prevent the obstruction of neurons in one plane by skin autofluorescence in adjacent planes, which would otherwise cover and hide them in the max-projection. **Top (A-D)**: max-projections of confocal images from forebrain and midbrain (A), midbrain and hindbrain (B), posterior hindbrain and spinal cord (C) and spinal cord (D), as shown in Fig. 2, before and after removal of the skin autofluorescence. **Bottom (E-F)**: Two examples of individual z-planes from the max-projection shown in (B), before and after removal of the skin autofluorescence. Scale bar = 50 µm.

bioRxiv preprint doi: https://doi.org/10.64898/2026.01.30.702415; this version posted January 31, 2026. The copyright holder for this preprint (which was not certified by peer review) is the author/funder, who has granted bioRxiv a license to display the preprint in perpetuity. It is made available under aCC-BY 4.0 International license.

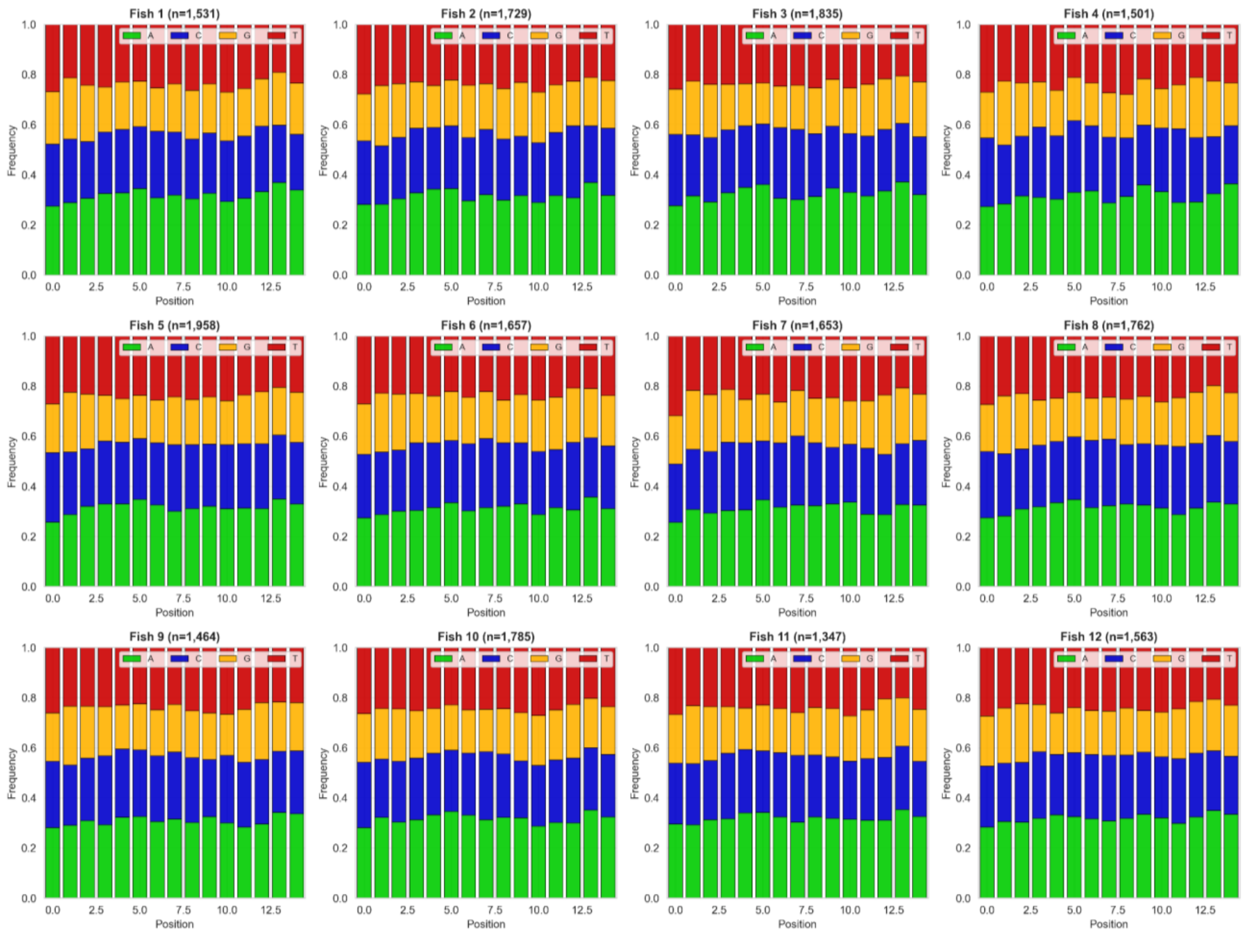

Figure S3 - Nucleotide composition for each position in the set of barcodes recovered from each fish, related to Fig. 3. DNA sequence logos showing the positional nucleotide frequencies across all unique barcodes recovered from each of the 12 individual fish. Only 15-nt barcodes were included in the frequency calculations, although an additional minority of barcodes were 14 or 16 nt long (<1%). For each position, the height of each colored segment represents the proportion of barcodes containing that nucleotide at that position. The number in parentheses in the subtitle for each plot indicates the total number of unique barcodes analyzed for that fish sample. The close to uniform nucleotide frequencies across all positions show a similar lack of sequence bias in the recovered barcode population for all the animals analyzed.

bioRxiv preprint doi: https://doi.org/10.64898/2026.01.30.702415; this version posted January 31, 2026. The copyright holder for this preprint (which was not certified by peer review) is the author/funder, who has granted bioRxiv a license to display the preprint in perpetuity. It is made available under aCC-BY 4.0 International license.

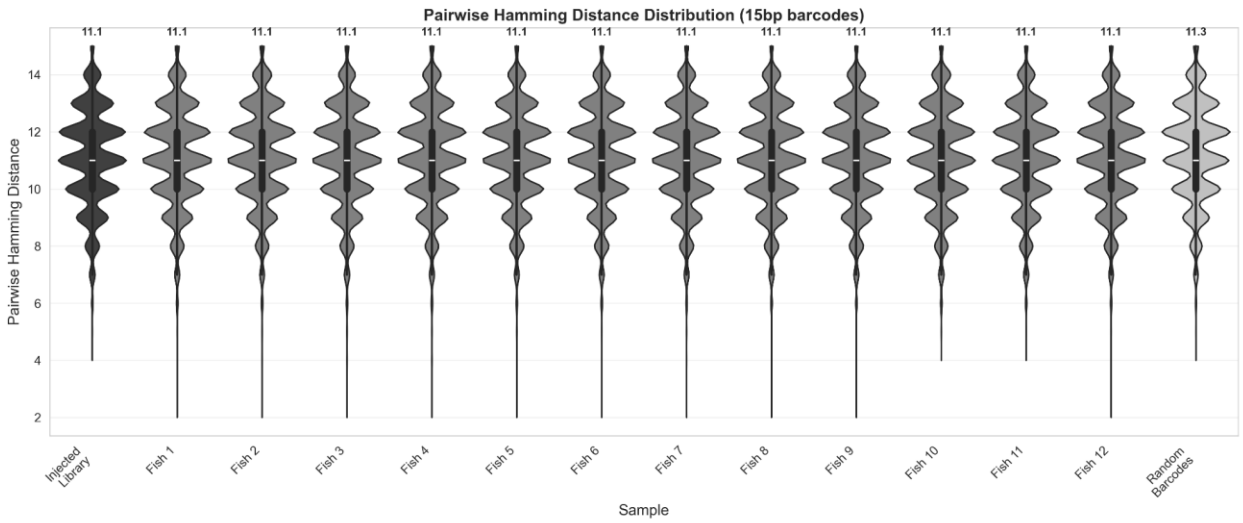

Figure S4 - Comparison of the distribution of pairwise Hamming distances for the sets of barcodes integrated in each fish, the original injected library, and a theoretical library of uniformly distributed random 15-nt barcodes. Distributions of pairwise distances are shown for 20,000 random pairs taken from each set. On top of each violin plot there is an overlaid boxplot, showing the median and interquantile range (IQR, representing 25th and 75th percentiles), and whiskers extending to 1.5×IQR beyond the quartiles. The number above each violin plot shows the mean Hamming distance in each sample. The narrow distribution centered around Hamming distance 11.1 (close to the theoretical maximum for random sequences) indicates that barcodes are highly dissimilar to each other, confirming minimal sequence clustering or bias in the samples. Furthermore, it confirms that the integrated barcodes from the mosaic fish retained the same sequence diversity as the original injected library.

bioRxiv preprint doi: https://doi.org/10.64898/2026.01.30.702415; this version posted January 31, 2026. The copyright holder for this preprint (which was not certified by peer review) is the author/funder, who has granted bioRxiv a license to display the preprint in perpetuity. It is made available under aCC-BY 4.0 International license.

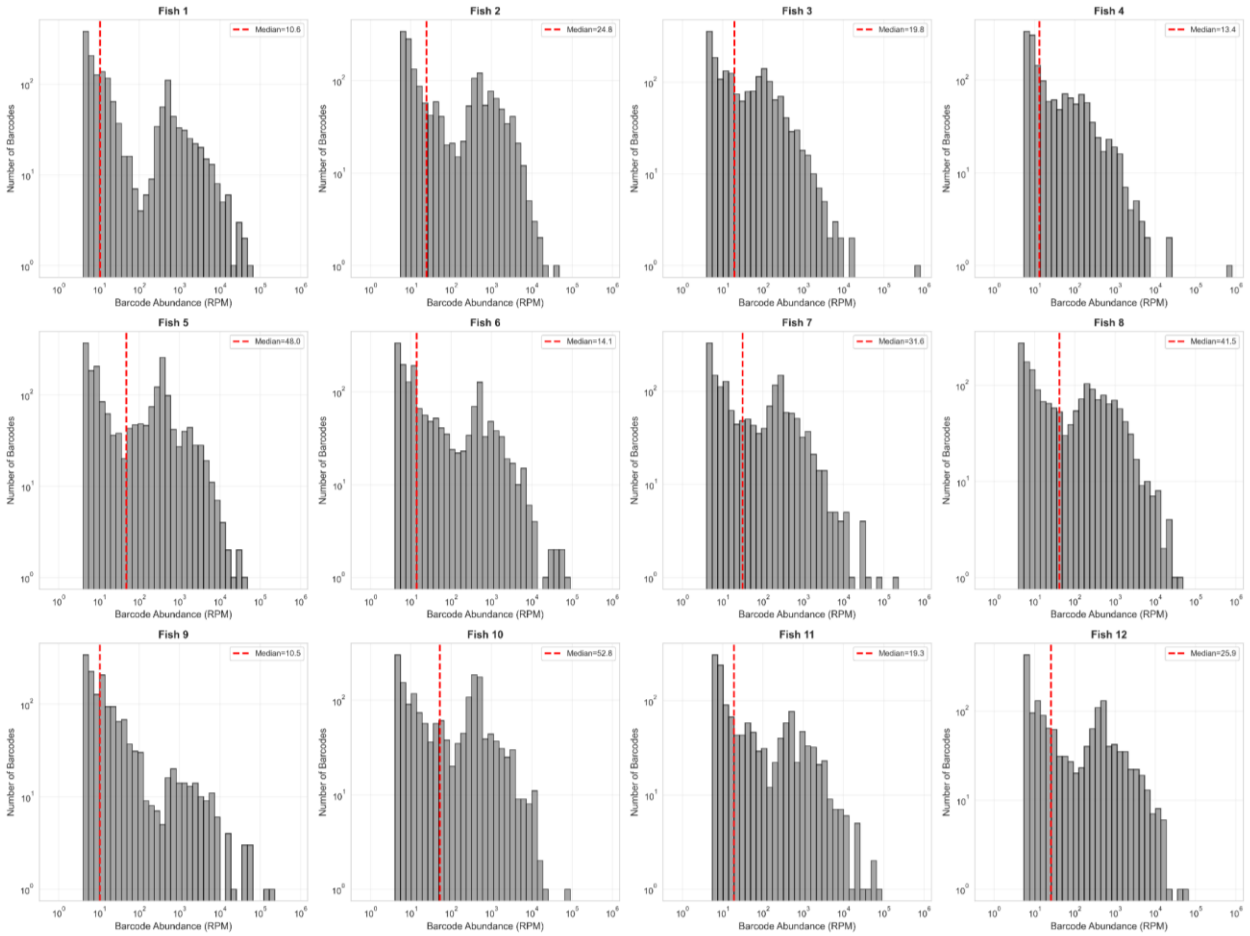

Figure S5 - Distribution of abundance for the set of barcodes recovered from each fish, related to Figure 3. RPM=reads per million.

bioRxiv preprint doi: https://doi.org/10.64898/2026.01.30.702415; this version posted January 31, 2026. The copyright holder for this preprint (which was not certified by peer review) is the author/funder, who has granted bioRxiv a license to display the preprint in perpetuity. It is made available under aCC-BY 4.0 International license.

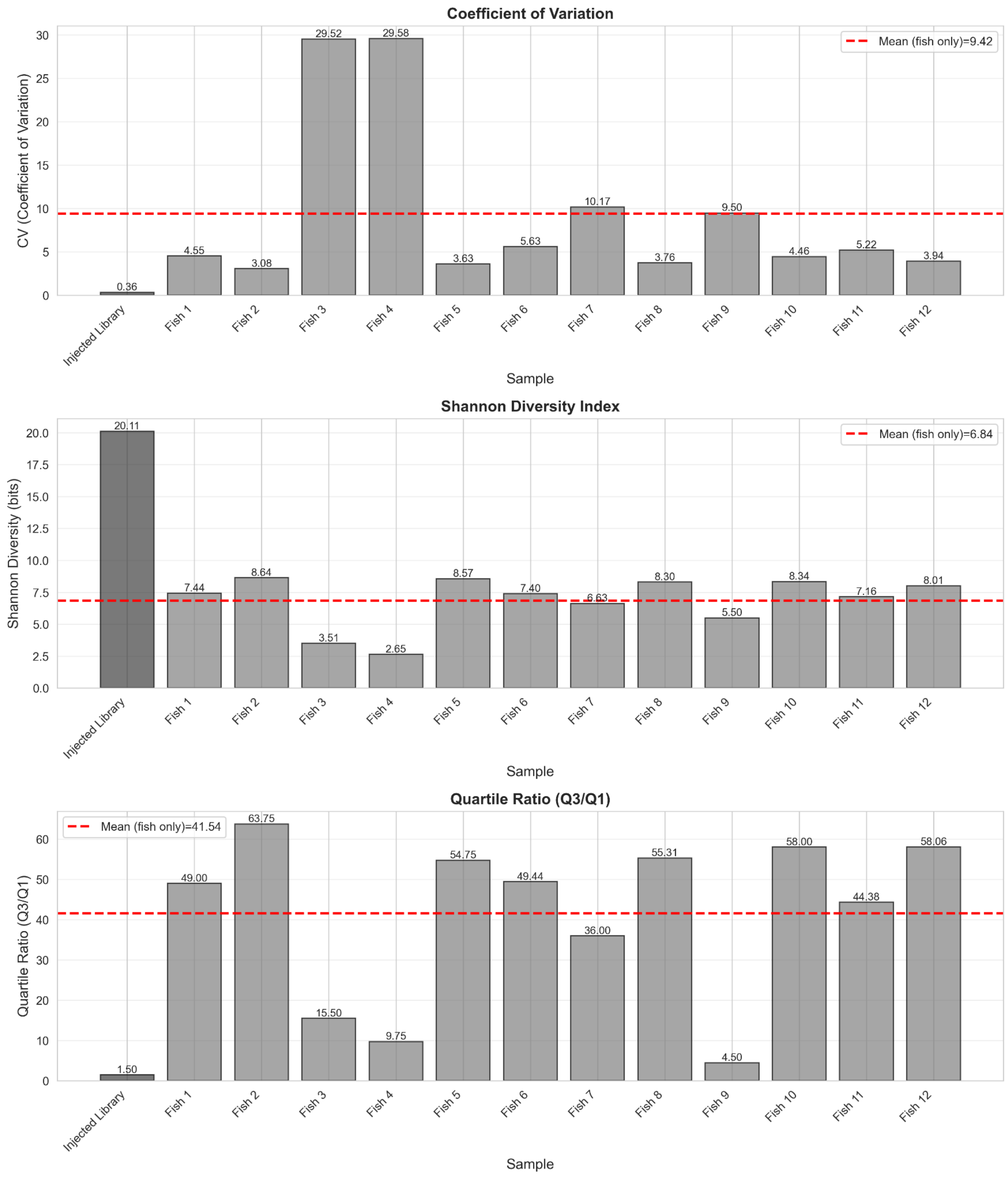

<u>Figure S6 -</u> Abundance distribution metrics for the injected library and integrated barcodes recovered from each fish, quantifying the extent of clonal expansion heterogeneity and the proliferative differences among cells that received different barcode integrations. The number above each bar plot shows the value for that sample. Overall, higher CV, lower Shannon diversity and higher quartile ratios show an increase in skewness of the abundance

bioRxiv preprint doi: https://doi.org/10.64898/2026.01.30.702415; this version posted January 31, 2026. The copyright holder for this preprint (which was not certified by peer review) is the author/funder, who has granted bioRxiv a license to display the preprint in perpetuity. It is made available under aCC-BY 4.0 International license.

distribution of the barcode represented in each fish, consistent with intra-fish clonal expansion of integrated barcodes.
The CV (coefficient of variation) is calculated as the standard deviation divided by mean of barcode read counts. A higher CV means some barcodes are much more abundant than others (uneven distribution), while a lower CV indicates more uniform barcode representation.Shannon diversity is an entropy-based metric that considers both the number of unique barcodes and the evenness of their distribution (in bits). It quantifies the overall barcode diversity, accounting for both richness (how many different barcodes) and evenness (how uniformly distributed their abundances are). Higher values indicate more diverse, more evenly distributed barcode populations. The quartile ratio is calculated as the ratio of the 75th percentile to the 25th percentile of barcode abundance, quantifying the spread of the middle 50% of barcode abundances. Higher ratios indicate greater inequality in barcode representation.

Table S1 - Ratio of multi-transgene neurons in the brains of mosaic zebrafish.
Quantification of neurons expressing GFP-only, mScarlet-only, or both fluorescent transgenes in mosaic transgenic zebrafish brains. **Z-plane:** The specific optical section(s) analyzed from the confocal Z-stack, indicated as the plane number out of the total stack depth (e.g., "30/64" means plane 30 from a 64-plane stack). Brain region(s) visible in each plane are indicated in parentheses. For Fish 7 and 8, the entire hindbrain volume was analyzed rather than a single plane. **GFP/mScarlet only:** Number of neurons expressing only the GFP or mScarlet transgene. **Both:** Number of neurons co-expressing both the GFP and mScarlet transgenes. **All:** Total number of transgene-positive neurons counted (GFP only + mScarlet only + Both). **Ratio (Both/All):** Percentage of all transgene-positive neurons that express both fluorophores, compared to all counted neurons. This ratio is used to estimate the frequency of multi-transgene integration events. **Combined**: Summary statistics pooling all the analyzed planes across all fish.

Fish ID	Age	pIGLET line	Z-plane	GFP only	mScarlet only	Both	All	Ratio (Both/All)
Fish 1	5 dpf	14a	30/64 (hindbrain and forebrain)	413	93	0	506	0.00%
Fish 1	5 dpf	14a	1/19 (spinal cord)	89	51	3	143	2.10%
Fish 2	5 dpf	14a	16/60 (hindbrain)	51	11	1	63	1.59%
Fish 2	5 dpf	14a	33/60 (hindbrain)	95	56	0	151	0.00%
Fish 3	5 dpf	14a	28/62 (hindbrain and forebrain)	189	57	1	247	0.40%
Fish 4	5 dpf	14a	12/79 (hindbrain and midbrain)	43	22	0	65	0.00%
Fish 4	5 dpf	14a	17/79 (hindbrain and midbrain)	56	41	1	98	1.02%
Fish 4	5 dpf	14a	23/79 (hindbrain	80	35	0	115	0.00%

bioRxiv preprint doi: https://doi.org/10.64898/2026.01.30.702415; this version posted January 31, 2026. The copyright holder for this preprint (which was not certified by peer review) is the author/funder, who has granted bioRxiv a license to display the preprint in perpetuity. It is made available under aCC-BY 4.0 International license.

			and midbrain)					
Fish 4	5 dpf	14a	30/79 (hindbrain and midbrain)	48	35	1	84	1.19%
Fish 5	5 dpf	14a	20/63 (hindbrain and midbrain)	30	15	0	45	0.00%
Fish 5	5 dpf	14a	25/63 (hindbrain and midbrain)	64	18	0	82	0.00%
Fish 6	3 dpf	24b	1 to 15/89 (forebrain)	79	36	0	115	0.00%
Fish 6	3 dpf	24b	36/89 (forebrain)	94	40	0	134	0.00%
Fish 6	3 dpf	24b	12/68 (hindbrain)	88	53	1	142	0.70%
Fish 6	3 dpf	24b	21/68 (hindbrain)	155	76	3	234	1.28%
Fish 6	3 dpf	24b	27/68 (hindbrain)	159	88	2	249	0.80%
Fish 7	5 dpf	14a	Entire hindbrain volume	748	222	10	980	1.02%
Fish 8	5 dpf	24b	Entire hindbrain volume	1030	491	10	1531	0.65%
**Combined:**				**3511**	**1440**	**33**	**4984**	**0.66%**

Table S2 - Sequencing library quality metrics. Quality metrics for the Illumina sequencing libraries from the injected barcode library and pooled integrated barcodes extracted from 12 individual fish. **Mean quality score**: Average Phred quality score across all base calls in the library. The Phred quality score is a logarithmic measure of base calling accuracy, calculated as $Q = -10 \times \log_{10}(P)$, where P is the probability of an incorrect base call. Q20: 1% error rate (99% accuracy), Q30: 0.1% error rate (99.9% accuracy). **Bases ≥ Q20**: Percentage of sequenced bases with Phred quality score of 20 or higher. **Bases ≥ Q30**: Percentage of sequenced bases with Phred quality score of 30 or higher.

	**Injected barcode library**	**Integrated barcodes (12 pooled fish samples)**
**Sequenced reads**	5,822,820	7,191,895
**Mean quality score:**	39.13	39.22
**Bases >= Q20:**	99.13%	99.02%
**Bases >= Q30:**	95.24%	95.84%

bioRxiv preprint doi: https://doi.org/10.64898/2026.01.30.702415; this version posted January 31, 2026. The copyright holder for this preprint (which was not certified by peer review) is the author/funder, who has granted bioRxiv a license to display the preprint in perpetuity. It is made available under aCC-BY 4.0 International license.

Table 3 - barcode counts and read retention throughout the stages of barcode extraction and processing. Reads, after demultiplexing: Total number of paired-end sequencing reads assigned to each sample. For fish samples, reads were demultiplexed based on 5bp sample barcodes at the read start, allowing up to 1 mismatch for error correction. The injected library was sequenced separately and required no demultiplexing. **Reads, after barcode extraction:** Number of reads from which valid barcodes were successfully extracted. Extraction required identifying conserved anchor sequences flanking the random barcode region. Reads lacking proper anchor sequences or with barcodes outside expected positions and lengths (14-16 nt) were discarded. **Reads, after barcode collapse:** Number of reads remaining after merging similar barcodes. Collapsing merges counts into parent barcodes but does not discard reads. **Reads, after barcode filtering:** Number of reads associated with barcodes that passed abundance and sequence filters. For the fish samples, barcodes were excluded from a fish if they appeared <3 times in that fish. For the injected library, barcodes with <2 reads in the injected library were excluded. Additionally, barcodes too similar (Levenshtein distance ≤2) to the conserved non-barcode regions of the injected plasmid were removed. **Barcodes, after extraction:** Number of unique barcode sequences identified after extraction, before any quality filtering or collapsing. **Barcodes, after collapsing:** Number of unique barcode sequences after collapsing. Barcodes within Levenshtein distance of 1, assumed to result from PCR or sequencing errors, were merged into their most abundant neighbor (parent barcode), with read counts combined. **Barcodes, after filtering:** Number of unique barcodes retained after applying the abundance and sequence filters described above.

Sample	Reads, after demultiplexing	Reads, after barcode extraction	Reads, after barcode collapse	Reads, after barcode filtering	Barcodes, after extraction	Barcodes, after collapsing	Barcodes, after filtering
Fish 1	604707	568573	568573	563410	8679	5933	1553
Fish 2	572589	528807	528807	524074	9234	5797	1761
Fish 3	567819	558715	558715	555185	6180	4699	1861
Fish 4	538318	525189	525189	521701	5600	4330	1524
Fish 5	643730	588909	588909	583426	10301	6749	1989
Fish 6	598294	573249	573249	568572	8574	5700	1682
Fish 7	598419	574577	574577	570243	7981	5383	1682
Fish 8	634168	608624	608624	603129	10129	6623	1792
Fish 9	584056	574166	574166	570225	6622	4728	1489

bioRxiv preprint doi: https://doi.org/10.64898/2026.01.30.702415; this version posted January 31, 2026. The copyright holder for this preprint (which was not certified by peer review) is the author/funder, who has granted bioRxiv a license to display the preprint in perpetuity. It is made available under aCC-BY 4.0 International license.

Fish 10	602081	553224	553224	548768	8974	5666	1810
Fish 11	552802	521451	521451	517234	7316	4937	1378
Fish 12	568603	545314	545314	540172	9187	6118	1592
Injected library	5822820	5499123	5499123	3018743	4176867	3670283	1190778

bioRxiv preprint doi: https://doi.org/10.64898/2026.01.30.702415; this version posted January 31, 2026. The copyright holder for this preprint (which was not certified by peer review) is the author/funder, who has granted bioRxiv a license to display the preprint in perpetuity. It is made available under aCC-BY 4.0 International license.

Appendix data S1: barcoded AttB-HS4-15N-nrUAS-GFP-CAAX plasmid sequence

Map:	AttB	HS4 insulator	DNA barcode	4xnrUAS	GFP-CAAX

AATACTCATACTCTTCCTTTTTCAATATTATTGAAGCATTTATCAGGGTTATTGTCTCATGAG
CGGATACATATTTGAATGTATTTAGAAAAATAAACAAATAGGGGTTCCGCGCACATTTCCCC
GAAAAGTGCCAGATACCTGAAACAAAACCCATCGTACGGCCAAGGAAGTCTCCAATAACTG
TGATCCACCACAAGCGCCAGGGTTTTCCCAGTCACGACGTTGTAAAACGACGGCCAGTCA
TGCATAATCCGCACGCATCTGGAATAAGGAAGTGCCATTCCGCCTGACCTCTCGAAGCCG
CGGTGCGGGTGCCAGGGCGTGCCCTTGGGCTCCCCGGGCGCGTACTCCACCTCACCCAT
CGAGCTCACGGGGACAGCCCCCTCCCAAAGCCCCCAGGGANNNNNNNNNNNNNNNCACG
CTAGCTGTAATTACGTCCCTCCCCCGCTAGGGGGCAGCAGCGAGCCGCCCGGGGCTCCG
CTCCGGTCCGGCGCTCCCCCCGCATCCCCGAGCCGGCAGCGTGCGGGGACAGCCCGGG
CACGGGGAAGGTGGCACGGGATCGCTTTCCTCTGAACGCTTCTCGCTGCTCTTTGAGCCT
GCAGACACCTGGGGGGATACGGGGAAAAAGCTTTAGGCTGAAAGAGAGATTTAGAATGAC
AGGCGCGCCACTAGTCGGTGGCTTCTAATCCGTGAGTCCTAGCGGGTGACAGCCCTCCGT
CTTCACAGGCGGAGGAGAGTCTTCCGTAGGGTTCCTCGGAGTACTGTCCTCCGACGCGTG
CAAGGGTCGACTCTAGAGGGTATATAATGGATCCCATCGCGTCTCAGCCTCACTTTGAGCT
CCTCCACACGCCACCATGGTTAGTAAAGGTGAGGAGCTGTTTACAGGTGTCGTGCCGATT
CTCGTGGAACTTGACGGCGATGTAAATGGGCATAAATTCAGCGTATCTGGGGAAGGTGAG
GGCGACGCAACTTACGGTAAACTGACCCTCAAGTTCATATGTACTACAGGGAAACTGCCTG
TGCCGTGGCCTACTCTGGTAACAACTTTGACGTATGGCGTCCAATGTTTTAGCCGATATCC
CGATCACATGAAACAACACGATTTCTTTAAATCAGCCATGCCTGAAGGATATGTGCAAGAA
CGAACCATTTTCTTCAAAGACGATGGCAATTATAAAACCCGTGCAGAGGTTAAGTTTGAGG
GCGATACACTCGTTAATCGGATCGAGCTGAAAGGAATAGACTTTAAGGAAGACGGCAATAT
TCTGGGGCATAAACTGGAGTATAATTACAATTCACACAATGTCTACATCATGGCAGATAAGC
AGAAGAACGGGATTAAAGTCAATTTCAAGATTAGACACAACATCGAAGACGGCTCCGTTCA
ACTCGCGGATCATTATCAGCAAAATACGCCCATCGGTGATGGCCCCGTTCTGCTCCCAGAT
AACCACTATTTGAGCACGCAGAGCGCACTGTCAAAGGACCCTAATGAGAAAAGAGATCATA
TGGTGCTCCTTGAGTTTGTTACAGCAGCTGGGATCACATTGGGGATGGATGAACTTTACAA
AAAGCTGAACCCTCCTGATGAGAGTGGCCCCGGCTGCATGAGCTGCAAGTGTGTGCTCTC
CTAAGATCCAGACATGATAAGATACATTGATGAGTTTGGACAAACCACAACTAGAATGCAGT
GAAAAAAATGCTTTATTTGTGAAATTTGTGATGCTATTGCTTTATTTGTAACCATTATAAGCT
GCAATAAACAAGTTAACAACAACAATTGCATTCATTTTATGTTTCAGGTTCAGGGGGAGGTG
TGGGAGGTTTTTTAAAGGCTAGGTGGAGGCTCAGTGATGATAAGTCTGCGATGGTGGATG
CATGTGTCATGGTCATAGCTGTTTCCTGTGTGAAATTGTTATCCGCTCAGAGGGCACAATC
CTATTCCGCGCTATCCGACAATCTCCAAGACATTAGGTGGAGTTCAGTTCGGCGTATGGCA
TATGTCGCTGGAAAGAACATGTGAGCAAAAGGCCAGCAAAAGGCCAGGAACCGTAAAAAG
GCCGCGTTGCTGGCGTTTTTCCATAGGCTCCGCCCCCCTGACGAGCATCACAAAAATCGA
CGCTCAAGTCAGAGGTGGCGAAACCCGACAGGACTATAAAGATACCAGGCGTTTCCCCCT
GGAAGCTCCCTCGTGCGCTCTCCTGTTCCGACCCTGCCGCTTACCGGATACCTGTCCGCC
TTTCTCCCTTCGGGAAGCGTGGCGCTTTCTCATAGCTCACGCTGTAGGTATCTCAGTTCGG
TGTAGGTCGTTCGCTCCAAGCTGGGCTGTGTGCACGAACCCCCCGTTCAGCCCGACCGCT
GCGCCTTATCCGGTAACTATCGTCTTGAGTCCAACCCGGTAAGACACGACTTATCGCCACT
GGCAGCAGCCACTGGTAACAGGATTAGCAGAGCGAGGTATGTAGGCGGTGCTACAGAGTT
CTTGAAGTGGTGGCCTAACTACGGCTACACTAGAAGAACAGTATTTGGTATCTGCGCTCTG
CTGAAGCCAGTTACCTTCGGAAAAAGAGTTGGTAGCTCTTGATCCGGCAAACAAACCACCG
CTGGTAGCGGTGGTTTTTTTGTTTGCAAGCAGCAGATTACGCGCAGAAAAAAAGGATCTCA
AGAAGATCCTTTGATCTTTTCTACGGGGTCTGACGCTCTATTCAACAAAGCCGCCGTCCCG
TCAAGTCAGCGTAAATGGGTAGGGGGCTTCAAATCGTCCTCGTGATACCAATTCGGAGCCT

bioRxiv preprint doi: https://doi.org/10.64898/2026.01.30.702415; this version posted January 31, 2026. The copyright holder for this preprint (which was not certified by peer review) is the author/funder, who has granted bioRxiv a license to display the preprint in perpetuity. It is made available under aCC-BY 4.0 International license.

GCTTTTTTGTACAAACTTGTTGATAATGGCAATTCAAGGATCTTCACCTAGATCCTTTTAAAT
TAAAAATGAAGTTTTAAATCAATCTAAAGTATATATGAGTAAACTTGGTCTGACAGTTACCAA
TGCTTAATCAGTGAGGCACCTATCTCAGCGATCTGTCTATTTCGTTCATCCATAGTTGCCTG
ACTCCCCGTCGTGTAGATAACTACGATACGGGAGGGCTTACCATCTGGCCCCAGTGCTGC
AATGATACCGCGAGAGCCACGCTCACCGGCTCCAGATTTATCAGCAATAAACCAGCCAGC
CGGAAGGGCCGAGCGCAGAAGTGGTCCTGCAACTTTATCCGCCTCCATCCAGTCTATTAA
TTGTTGCCGGGAAGCTAGAGTAAGTAGTTCGCCAGTTAATAGTTTGCGCAACGTTGTTGCC
ATTGCTACAGGCATCGTGGTGTCACGCTCGTCGTTTGGTATGGCTTCATTCAGCTCCGGTT
CCCAACGATCAAGGCGAGTTACATGATCCCCCATGTTGTGCAAAAAAGCGGTTAGCTCCTT
CGGTCCTCCGATCGTTGTCAGAAGTAAGTTGGCCGCAGTGTTATCACTCATGGTTATGGCA
GCACTGCATAATTCTCTTACTGTCATGCCATCCGTAAGATGCTTTTCTGTGACTGGTGAGTA
CTCAACCAAGTCATTCTGAGAATAGTGTATGCGGCGACCGAGTTGCTCTTGCCCGGCGTC
AATACGGGATAATACCGCGCCACATAGCAGAACTTTAAAAGTGCTCATCATTGGAAAACGT
TCTTCGGGGCGAAAACTCTCAAGGATCTTACCGCTGTTGAGATCCAGTTCGATGTAACCCA
CTCGTGCACCCAACTGATCTTCAGCATCTTTTACTTTCACCAGCGTTTCTGGGTGAGCAAAA
ACAGGAAGGCAAAATGCCGCAAAAAAGGGAATAAGGGCGACACGGAAATGTTG

Appendix data S2: AttB-HS4-nrUAS-GFP-CAAX plasmid sequence

CGACGTTGTAAAACGACGGCCAGTCATGCATAATCCGCACGCATCTGGAATAAGGAAGTG
CCATTCCGCCTGACCTCTCGAAGCCGCGGTGCGGGTGCCAGGGCGTGCCCTTGGGCTCC
CCGGGCGCGTACTCCACCTCACCCATCGAGCTCACGGGGACAGCCCCCTCCCAAAGCCC
CCAGGGATGTAATTACGTCCCTCCCCCGCTAGGGGGCAGCAGCGAGCCGCCCGGGGCTC
CGCTCCGGTCCGGCGCTCCCCCCGCATCCCCGAGCCGGCAGCGTGCGGGGACAGCCCG
GGCACGGGGAAGGTGGCACGGGATCGCTTTCCTCTGAACGCTTCTCGCTGCTCTTTGAGC
CTGCAGACACCTGGGGGGATACGGGGAAAAAGCTTTAGGCTGAAAGAGAGATTTAGAATG
ACAGGCGCGCCACTAGTCGGTGGCTTCTAATCCGTGAGTCCTAGCGGGTGACAGCCCTCC
GTCTTCACAGGCGGAGGAGAGTCTTCCGTAGGGTTCCTCGGAGTACTGTCCTCCGACGCG
TGCAAGGGTCGACTCTAGAGGGTATATAATGGATCCCATCGCGTCTCAGCCTCACTTTGAG
CTCCTCCACACGCCACCATGGTTAGTAAAGGTGAGGAGCTGTTTACAGGTGTCGTGCCGA
TTCTCGTGGAACTTGACGGCGATGTAAATGGGCATAAATTCAGCGTATCTGGGGAAGGTGA
GGGCGACGCAACTTACGGTAAACTGACCCTCAAGTTCATATGTACTACAGGGAAACTGCCT
GTGCCGTGGCCTACTCTGGTAACAACTTTGACGTATGGCGTCCAATGTTTTAGCCGATATC
CCGATCACATGAAACAACACGATTTCTTTAAATCAGCCATGCCTGAAGGATATGTGCAAGA
ACGAACCATTTTCTTCAAAGACGATGGCAATTATAAAACCCGTGCAGAGGTTAAGTTTGAG
GGCGATACACTCGTTAATCGGATCGAGCTGAAAGGAATAGACTTTAAGGAAGACGGCAATA
TTCTGGGGCATAAACTGGAGTATAATTACAATTCACACAATGTCTACATCATGGCAGATAAG
CAGAAGAACGGGATTAAAGTCAATTTCAAGATTAGACACAACATCGAAGACGGCTCCGTTC
AACTCGCGGATCATTATCAGCAAAATACGCCCATCGGTGATGGCCCCGTTCTGCTCCCAGA
TAACCACTATTTGAGCACGCAGAGCGCACTGTCAAAGGACCCTAATGAGAAAAGAGATCAT
ATGGTGCTCCTTGAGTTTGTTACAGCAGCTGGGATCACATTGGGGATGGATGAACTTTACA
AAAAGCTGAACCCTCCTGATGAGAGTGGCCCCGGCTGCATGAGCTGCAAGTGTGTGCTCT
CCTAAGATCCAGACATGATAAGATACATTGATGAGTTTGGACAAACCACAACTAGAATGCA
GTGAAAAAAATGCTTTATTTGTGAAATTTGTGATGCTATTGCTTTATTTGTAACCATTATAAG
CTGCAATAAACAAGTTAACAACAACAATTGCATTCATTTTATGTTTCAGGTTCAGGGGGAGG
TGTGGGAGGTTTTTTAAAGGCTAGGTGGAGGCTCAGTGATGATAAGTCTGCGATGGTGGA
TGCATGTGTCATGGTCATAGCTGTTTCCTGTGTGAAATTGTTATCCGCTCAGAGGGCACAA
TCCTATTCCGCGCTATCCGACAATCTCCAAGACATTAGGTGGAGTTCAGTTCGGCGTATGG
CATATGTCGCTGGAAAGAACATGTGAGCAAAAGGCCAGCAAAAGGCCAGGAACCGTAAAA

bioRxiv preprint doi: https://doi.org/10.64898/2026.01.30.702415; this version posted January 31, 2026. The copyright holder for this preprint (which was not certified by peer review) is the author/funder, who has granted bioRxiv a license to display the preprint in perpetuity. It is made available under aCC-BY 4.0 International license.

AGGCCGCGTTGCTGGCGTTTTTCCATAGGCTCCGCCCCCCTGACGAGCATCACAAAAATC
GACGCTCAAGTCAGAGGTGGCGAAACCCGACAGGACTATAAAGATACCAGGCGTTTCCCC
CTGGAAGCTCCCTCGTGCGCTCTCCTGTTCCGACCCTGCCGCTTACCGGATACCTGTCCG
CCTTTCTCCCTTCGGGAAGCGTGGCGCTTTCTCATAGCTCACGCTGTAGGTATCTCAGTTC
GGTGTAGGTCGTTCGCTCCAAGCTGGGCTGTGTGCACGAACCCCCCGTTCAGCCCGACC
GCTGCGCCTTATCCGGTAACTATCGTCTTGAGTCCAACCCGGTAAGACACGACTTATCGCC
ACTGGCAGCAGCCACTGGTAACAGGATTAGCAGAGCGAGGTATGTAGGCGGTGCTACAGA
GTTCTTGAAGTGGTGGCCTAACTACGGCTACACTAGAAGAACAGTATTTGGTATCTGCGCT
CTGCTGAAGCCAGTTACCTTCGGAAAAAGAGTTGGTAGCTCTTGATCCGGCAAACAAACCA
CCGCTGGTAGCGGTGGTTTTTTTGTTTGCAAGCAGCAGATTACGCGCAGAAAAAAAGGATC
TCAAGAAGATCCTTTGATCTTTTCTACGGGGTCTGACGCTCTATTCAACAAAGCCGCCGTC
CCGTCAAGTCAGCGTAAATGGGTAGGGGGCTTCAAATCGTCCTCGTGATACCAATTCGGA
GCCTGCTTTTTTGTACAAACTTGTTGATAATGGCAATTCAAGGATCTTCACCTAGATCCTTTT
AAATTAAAAATGAAGTTTTAAATCAATCTAAAGTATATATGAGTAAACTTGGTCTGACAGTTA
CCAATGCTTAATCAGTGAGGCACCTATCTCAGCGATCTGTCTATTTCGTTCATCCATAGTTG
CCTGACTCCCCGTCGTGTAGATAACTACGATACGGGAGGGCTTACCATCTGGCCCCAGTG
CTGCAATGATACCGCGAGAGCCACGCTCACCGGCTCCAGATTTATCAGCAATAAACCAGC
CAGCCGGAAGGGCCGAGCGCAGAAGTGGTCCTGCAACTTTATCCGCCTCCATCCAGTCTA
TTAATTGTTGCCGGGAAGCTAGAGTAAGTAGTTCGCCAGTTAATAGTTTGCGCAACGTTGT
TGCCATTGCTACAGGCATCGTGGTGTCACGCTCGTCGTTTGGTATGGCTTCATTCAGCTCC
GGTTCCCAACGATCAAGGCGAGTTACATGATCCCCCATGTTGTGCAAAAAAGCGGTTAGCT
CCTTCGGTCCTCCGATCGTTGTCAGAAGTAAGTTGGCCGCAGTGTTATCACTCATGGTTAT
GGCAGCACTGCATAATTCTCTTACTGTCATGCCATCCGTAAGATGCTTTTCTGTGACTGGT
GAGTACTCAACCAAGTCATTCTGAGAATAGTGTATGCGGCGACCGAGTTGCTCTTGCCCG
GCGTCAATACGGGATAATACCGCGCCACATAGCAGAACTTTAAAAGTGCTCATCATTGGAA
AACGTTCTTCGGGGCGAAAACTCTCAAGGATCTTACCGCTGTTGAGATCCAGTTCGATGTA
ACCCACTCGTGCACCCAACTGATCTTCAGCATCTTTTACTTTCACCAGCGTTTCTGGGTGA
GCAAAAACAGGAAGGCAAAATGCCGCAAAAAAGGGAATAAGGGCGACACGGAAATGTTGA
ATACTCATACTCTTCCTTTTTCAATATTATTGAAGCATTTATCAGGGTTATTGTCTCATGAGC
GGATACATATTTGAATGTATTTAGAAAAATAAACAAATAGGGGTTCCGCGCACATTTCCCCG
AAAAGTGCCAGATACCTGAAACAAAACCCATCGTACGGCCAAGGAAGTCTCCAATAACTGT
GATCCACCACAAGCGCCAGGGTTTTCCCAGTCA

Appendix data S3: AttB-HS4-nrUAS-mScarlet-CAAX plasmid sequence

CGACGTTGTAAAACGACGGCCAGTCATGCATAATCCGCACGCATCTGGAATAAGGAAGTG
CCATTCCGCCTGACCTCTCGAAGCCGCGGTGCGGGTGCCAGGGCGTGCCCTTGGGCTCC
CCGGGCGCGTACTCCACCTCACCCATCGAGCTCACGGGGACAGCCCCCTCCCAAAGCCC
CCAGGGATGTAATTACGTCCCTCCCCCGCTAGGGGGCAGCAGCGAGCCGCCCGGGGCTC
CGCTCCGGTCCGGCGCTCCCCCCGCATCCCCGAGCCGGCAGCGTGCGGGGACAGCCCG
GGCACGGGGAAGGTGGCACGGGATCGCTTTCCTCTGAACGCTTCTCGCTGCTCTTTGAGC
CTGCAGACACCTGGGGGGATACGGGGAAAAAGCTTTAGGCTGAAAGAGAGATTTAGAATG
ACAGGCGCGCCACTAGTCGGTGGCTTCTAATCCGTGAGTCCTAGCGGGTGACAGCCCTCC
GTCTTCACAGGCGGAGGAGAGTCTTCCGTAGGGTTCCTCGGAGTACTGTCCTCCGACGCG
TGCAAGGGTCGACTCTAGAGGGTATATAATGGATCCCATCGCGTCTCAGCCTCACTTTGAG
CTCCTCCACACGCCACCATGGTGAGCAAGGGCGAGGCAGTGATCAAGGAGTTCATGCGGT
TCAAGGTGCACATGGAGGGCTCCATGAACGGCCACGAGTTCGAGATCGAGGGCGAGGGC
GAGGGCCGCCCCTACGAGGGCACCCAGACCGCCAAGCTGAAGGTGACCAAGGGTGGCC
CCCTGCCCTTCTCCTGGGACATCCTGTCCCCTCAGTTCATGTACGGCTCCAGGGCCTTCA

bioRxiv preprint doi: https://doi.org/10.64898/2026.01.30.702415; this version posted January 31, 2026. The copyright holder for this preprint (which was not certified by peer review) is the author/funder, who has granted bioRxiv a license to display the preprint in perpetuity. It is made available under aCC-BY 4.0 International license.

CCAAGCACCCCGCCGACATCCCCGACTACTATAAGCAGTCCTTCCCCGAGGGCTTCAAGT
GGGAGCGCGTGATGAACTTCGAGGACGGCGGCGCCGTGACCGTGACCCAGGACACCTCC
CTGGAGGACGGCACCCTGATCTACAAGGTGAAGCTCCGCGGCACCAACTTCCCTCCTGAC
GGCCCCGTAATGCAGAAGAAGACAATGGGCTGGGAAGCGTCCACCGAGCGGTTGTACCC
CGAGGACGGCGTGCTGAAGGGCGACATTAAGATGGCCCTGCGCCTGAAGGACGGCGGCC
GATACCTGGCGGACTTCAAGACCACCTACAAGGCCAAGAAGCCCGTGCAGATGCCCGGC
GCCTACAACGTGGACCGCAAGTTGGACATCACCTCCCACAACGAGGACTACACCGTGGTG
GAACAGTACGAACGCTCCGAGGGCCGCCACTCCACCGGCGGCATGGACGAGCTGTACAA
GAAGCTGAACCCTCCTGATGAGAGTGGCCCCGGCTGCATGAGCTGCAAGTGTGTGCTCTC
CTAAGATCCAGACATGATAAGATACATTGATGAGTTTGGACAAACCACAACTAGAATGCAGT
GAAAAAAATGCTTTATTTGTGAAATTTGTGATGCTATTGCTTTATTTGTAACCATTATAAGCT
GCAATAAACAAGTTAACAACAACAATTGCATTCATTTTATGTTTCAGGTTCAGGGGGAGGTG
TGGGAGGTTTTTTAAAGGCTAGGTGGAGGCTCAGTGATGATAAGTCTGCGATGGTGGATG
CATGTGTCATGGTCATAGCTGTTTCCTGTGTGAAATTGTTATCCGCTCAGAGGGCACAATC
CTATTCCGCGCTATCCGACAATCTCCAAGACATTAGGTGGAGTTCAGTTCGGCGTATGGCA
TATGTCGCTGGAAAGAACATGTGAGCAAAAGGCCAGCAAAAGGCCAGGAACCGTAAAAAG
GCCGCGTTGCTGGCGTTTTTCCATAGGCTCCGCCCCCCTGACGAGCATCACAAAAATCGA
CGCTCAAGTCAGAGGTGGCGAAACCCGACAGGACTATAAAGATACCAGGCGTTTCCCCCT
GGAAGCTCCCTCGTGCGCTCTCCTGTTCCGACCCTGCCGCTTACCGGATACCTGTCCGCC
TTTCTCCCTTCGGGAAGCGTGGCGCTTTCTCATAGCTCACGCTGTAGGTATCTCAGTTCGG
TGTAGGTCGTTCGCTCCAAGCTGGGCTGTGTGCACGAACCCCCCGTTCAGCCCGACCGCT
GCGCCTTATCCGGTAACTATCGTCTTGAGTCCAACCCGGTAAGACACGACTTATCGCCACT
GGCAGCAGCCACTGGTAACAGGATTAGCAGAGCGAGGTATGTAGGCGGTGCTACAGAGTT
CTTGAAGTGGTGGCCTAACTACGGCTACACTAGAAGAACAGTATTTGGTATCTGCGCTCTG
CTGAAGCCAGTTACCTTCGGAAAAAGAGTTGGTAGCTCTTGATCCGGCAAACAAACCACCG
CTGGTAGCGGTGGTTTTTTTGTTTGCAAGCAGCAGATTACGCGCAGAAAAAAAGGATCTCA
AGAAGATCCTTTGATCTTTTCTACGGGGTCTGACGCTCTATTCAACAAAGCCGCCGTCCCG
TCAAGTCAGCGTAAATGGGTAGGGGGCTTCAAATCGTCCTCGTGATACCAATTCGGAGCCT
GCTTTTTTGTACAAACTTGTTGATAATGGCAATTCAAGGATCTTCACCTAGATCCTTTTAAAT
TAAAAATGAAGTTTTAAATCAATCTAAAGTATATATGAGTAAACTTGGTCTGACAGTTACCAA
TGCTTAATCAGTGAGGCACCTATCTCAGCGATCTGTCTATTTCGTTCATCCATAGTTGCCTG
ACTCCCCGTCGTGTAGATAACTACGATACGGGAGGGCTTACCATCTGGCCCCAGTGCTGC
AATGATACCGCGAGAGCCACGCTCACCGGCTCCAGATTTATCAGCAATAAACCAGCCAGC
CGGAAGGGCCGAGCGCAGAAGTGGTCCTGCAACTTTATCCGCCTCCATCCAGTCTATTAA
TTGTTGCCGGGAAGCTAGAGTAAGTAGTTCGCCAGTTAATAGTTTGCGCAACGTTGTTGCC
ATTGCTACAGGCATCGTGGTGTCACGCTCGTCGTTTGGTATGGCTTCATTCAGCTCCGGTT
CCCAACGATCAAGGCGAGTTACATGATCCCCCATGTTGTGCAAAAAAGCGGTTAGCTCCTT
CGGTCCTCCGATCGTTGTCAGAAGTAAGTTGGCCGCAGTGTTATCACTCATGGTTATGGCA
GCACTGCATAATTCTCTTACTGTCATGCCATCCGTAAGATGCTTTTCTGTGACTGGTGAGTA
CTCAACCAAGTCATTCTGAGAATAGTGTATGCGGCGACCGAGTTGCTCTTGCCCGGCGTC
AATACGGGATAATACCGCGCCACATAGCAGAACTTTAAAAGTGCTCATCATTGGAAAACGT
TCTTCGGGGCGAAAACTCTCAAGGATCTTACCGCTGTTGAGATCCAGTTCGATGTAACCCA
CTCGTGCACCCAACTGATCTTCAGCATCTTTTACTTTCACCAGCGTTTCTGGGTGAGCAAAA
ACAGGAAGGCAAAATGCCGCAAAAAAGGGAATAAGGGCGACACGGAAATGTTGAATACTC
ATACTCTTCCTTTTTCAATATTATTGAAGCATTTATCAGGGTTATTGTCTCATGAGCGGATAC
ATATTTGAATGTATTTAGAAAAATAAACAAATAGGGGTTCCGCGCACATTTCCCCGAAAAGT
GCCAGATACCTGAAACAAAACCCATCGTACGGCCAAGGAAGTCTCCAATAACTGTGATCCA
CCACAAGCGCCAGGGTTTTCCCAGTCA

bioRxiv preprint doi: https://doi.org/10.64898/2026.01.30.702415; this version posted January 31, 2026. The copyright holder for this preprint (which was not certified by peer review) is the author/funder, who has granted bioRxiv a license to display the preprint in perpetuity. It is made available under aCC-BY 4.0 International license.

Appendix data S4: Sequences of key primers used for library generation and sequencing

Primer sequence	Primer name	Use
GTTGATCATACATTGGCACGGCTAGCTGTAATTACGTCCCTCCCCCGCTA	F_HS4_NheI	2-cycle PCR to add random 15xN barcodes in the middle of the HS4 element for the plasmid library. An NheI restriction site is also added with these primers to enable specific sticky-end restriction-ligation for recircularization of the plasmid.
AGTCAAGTGGAATACTGCTAGCGTGNNNNNNNNNNNNNNNTCCCTGGGGGCTTTGGGAGG	R_Add15N_HS4_NheI	
CTTACTCATACATTGGCACGGC	F_amp_HS4	Amplification of the barcoded linearized whole-plasmid amplicons
AGTCAAGTGGAATACTGCTAGCG	R_amp_HS4	
ACAACCCGACAGCCTACGTCAC	F_Chr24pIGLET	Specific amplification of integrated barcodes from genomic extracts of mosaic zebrafish, generating a 652 bp amplicon library
GAGAAGCGTTCAGAGGAAAGCGATC	R_HS4	
TCGTCGGCAGCGTCAGATGTGTATAAGAGACAGTGGAGATCACTTCATTCTATTTTCCCT	F_Chr24_illumread	Generation of 325 bp amplicon library of fish-recovered integrated barcodes for direct Illumina sequencing, based on amplification of the 652 bp amplicon library and addition of Illumina overhangs and sample-specific 5-nt barcode for demultiplexing
GTCTCGTGGGCTCGGAGATGTGTATAAGAGACAGNNNNNTAGCGGGGGAGGGACGTAATT	R_HS4_FishX_illumread	
TCGTCGGCAGCGTCAGATGTGTATAAGAGACAGACGGGGACAGCCCCCTCCCAAAG	F_HS4_illumread	Generation of 336 bp amplicon library of barcodes from the injected plasmid library for direct Illumina sequencing
GTCTCGTGGGCTCGGAGATGTGTATAAGAGACAGCAGCCTAAAGCTTTTTCCCCGTATCC	R_HS4_illumread	
